# Mental health strain due to COVID-19: Psychological distress and somatic symptoms among health care workers and the general population in Kashmir, India

**DOI:** 10.1016/j.virusres.2026.199753

**Published:** 2026-05-22

**Authors:** Mutha Manzoor, Muddasir Sharief Banday, Bilal Ahmad Para, Vanitha Innocent Rani, Umme Hani, Subi Mol Raja, Sumaila Parveen, S. Ilambharathi, M Kiruthika, Jonaid Ahmad Malik

**Affiliations:** a Department of Clinical Pharmacology, Sher-i-Kashmir Institute of Medical Sciences, Soura 190011, Jammu & Kashmir, India; b Department of Clinical Research, Cutis Institute of Dermatology, Srinagar 190014, Jammu & Kashmir, India; c Department of Mathematical Sciences, Islamic University of Science and Technology, Awantipora 192122, Jammu & Kashmir, India; d Department of Community & Psychiatric Nursing, College of Nursing, King Khalid University, Muhayil, 61421 Asir Region, Saudi Arabia; e Department of Pharmaceutics, College of Pharmacy, King Khalid University, Abha, 61421, Saudi Arabia; f Department of Quality Control, Burjeel Hospital, 7400, Abu Dhabi, United Arab Emirates; g Saveetha College of Nursing, Saveetha University, Thandalam, Tamil Nadu 602105, India; h Department of Basic Medical Sciences, College of Applied Medical Sciences, King Khalid University, Muhayil, 61421, Asir, Saudi Arabia; i Vinayaka Mission Research Foundation, Salem, Tamil Nadu, India; j Department of Biomedical Engineering, Indian Institute of Technology, Ropar, India; k Department of Medical Microbiology, Cell Biology and Immunology, SIU School of Medicine, Springfield, IL, USA

**Keywords:** COVID-19, DASS-21, Quarantine, Anxiety, Depression, Stress

## Abstract

**Background/Objectives:**

The COVID-19 pandemic precipitated a global rise in psychological morbidities, markedly increasing depression, anxiety, and stress. This study provides early region-specific evidence from Kashmir on the post-pandemic mental health burden. Building on our prior findings of substantial psychological distress in this cohort, we examined associations between specific COVID-19 symptom profiles, symptom duration, hospitalization status, and the severity of depression, anxiety, and stress. Correlates of psychological distress were also identified. While this study uses the same cohort as our primary study it addresses a distinct research question not previously examined, and all novel findings (symptom-level associations, odds ratios from multivariable regression) are presented here for the first time.

**Methods:**

Psychological distress was assessed using a self-designed, validated DASS-21 questionnaire among 250 participants.

**Results:**

Overall prevalence rates were 54.8% for depression, 68.0% for anxiety, and 34.4% for stress. Among healthcare professionals (n = 161; 64.4%), extremely severe anxiety (21.1%), moderate depression (15.5%), and mild-to-moderate stress (11.8%) predominated. In the community group (n = 89; 35.6%), moderate depression (33.7%), moderate anxiety (23.6%), and moderate stress (16.9%) were most frequent. Significant associations were observed between psychological distress and symptoms including fever (OR=4.2; 95% CI: 1.8–10.1), breathlessness (OR=3.2; 95% CI: 1.5–6.9), and decreased level of consciousness (OR=11.4; 95% CI: 1.1–109.2). Symptom duration ≥15 days was also significantly associated with higher distress levels (p < 0.05).

**Conclusion:**

A high prevalence of depression, anxiety, and stress was observed, particularly among individuals with symptomatic and prolonged COVID-19 illness. Symptom severity and extended disease duration was significantly associated to psychological distress, underscoring the need for targeted mental health interventions in post-pandemic settings.

## Highlights

• A high burden of psychological distress was observed in a Kashmiri cohort post-COVID-19, with prevalence rates of 54.8% for depression, 68.0% for anxiety, and 34.4% for stress.
• Specific COVID-19 symptoms were significant predictors of mental health outcomes: fever (OR=4.2), breathlessness (OR=3.2), and decreased level of consciousness (OR=11.4) showed the strongest associations with DASS-21 scores.
• Prolonged illness duration (≥15 days) was significantly associated with higher scores for depression, anxiety, and stress, highlighting the psychological toll of extended sickness.
• Despite severe clinical presentations, hospitalization status alone was not a significant predictor of psychological distress in this cohort, suggesting that symptom severity, rather than the care setting, may be a key driver.
• The findings underscore the urgent need for integrated mental health screening and targeted interventions for COVID-19 patients presenting with severe or persistent physical symptoms.

## 1 Introduction

On March 11, 2020, the World Health Organization (WHO) officially declared the Coronavirus disease 2019 (COVID-19) outbreak a global pandemic (D. et al., 2020). This public health crisis amplified beyond a profound threat to physical health, precipitating a parallel and silent mental health pandemic worldwide (Kunzler et al., 2021). Epidemiological data from the period indicate a significant escalation in the global burden of mental disorders, marked by an approximate 16% rise in incidence rates and a 12.5% increase in overall health loss, as measured by standardized metrics. Major depressive disorder and anxiety disorders were identified as the most prevalent conditions contributing to this increased burden. The frequency of these disorders demonstrated a significant gender disparity, with females constituting an overly affected demographic. The mental health burden was intensified by a confluence of pandemic-related factors, including widespread social isolation, economic insecurity, disruptions to healthcare services, and heightened population-level psychological distress (Global, regional, and national burden of 12 mental disorders in 204 countries and territories, 2022). Critically, the impact of the pandemic on global mental health was not uniform; its effects were differentially distributed across populations, with socioeconomic status emerging as a key determinant of vulnerability and outcomes (Moeti et al., al.).

The COVID-19 pandemic posed a gruesome picture in India, a developing nation with substantial vulnerable populations (children, the elderly, and women) and a healthcare system already operating under considerable strain. In this context, managing the pandemic placed extraordinary demands on medical infrastructure, diverting attention and resources away from routine health issues. Consequently, mental health concerns were largely overshadowed, despite their escalating significance (Roy et al., 2021). The psychological impact of COVID-19 in India has been profound and enduring, exacerbated by overwhelmed health services and widespread mortality. Public health measures such as quarantine and social isolation precipitated loneliness and sleep disturbances, while economic instability and employment loss generated pervasive uncertainty. Further compounding this crisis, reports of heightened domestic violence and abuse emerged as significant triggers of psychological distress. Projections of the mental health consequences of COVID-19 in India are deeply concerning (Kumar and Nayar, 2020), with the Indian Psychiatry Society documenting a 20% surge in mental illnesses since the pandemic's onset. Addressing this escalating mental health crisis is critically challenging in a setting of severe workforce shortages: India has only 0.29 psychiatrists, 0.07 psychologists, and 0.36 other mental health workers per 100,000 people (Ray, 2022). These systemic inadequacies, coupled with persistent stigma, limited public awareness, and insufficient sustainable care models, have long contributed to a substantial treatment gap. Prior to the pandemic, India reported an estimated 197.3 million individuals with mental disorders, including 46 million with depression and 45 million with anxiety. The National Mental Health Survey highlighted a treatment gap of 83%, starkly higher than the 40% observed in high-income countries. Another pre-pandemic study indicated that as few as one in ten affected individuals seek mental healthcare annually, suggesting a treatment gap as high as 95% (Article, 2025).

While initial studies, including our prior analysis of this Kashmiri cohort, have established a high prevalence of depression, anxiety, and stress among both healthcare workers and the general population (Sharief Banday et al., 2023) a critical gap remains. The specific clinical correlates of this psychological distress, particularly the role of specific COVID-19 symptoms, the duration of illness, and hospitalization status, are yet to be elucidated in this region. Understanding these correlates is vital, as they may serve as predictive markers for long-term mental health sequelae, enabling early intervention and resource allocation for high-risk groups.

In our primary study using this cohort (Sharief Banday et al., 2023), we reported a high prevalence of depression (54.8%), anxiety (68.0%), and stress (34.4%) and examined associations with sociodemographic variables. However, that study did not and was not designed to investigate associations with specific COVID-19 symptoms, symptom duration, or hospitalization. Adding these extensive analyses to the primary study would have resulted in an excessively long manuscript, potentially obscuring the main findings for readers. Therefore, we elected to conduct the present secondary analysis as a separate, focused study addressing a distinct and clinically important question: Are specific COVID-19 symptom profiles associated with greater psychological distress? Identifying such associations could help target mental health screening to high-risk individuals.Therefore, this secondary analysis extends our initial work with the primary objective of determining the correlation between specific COVID-19 clinical features (symptoms, illness duration, and hospitalization) and the prevalence of depression, anxiety, and stress. By identifying these modifiable risk factors, this study aims to contribute critical evidence to inform targeted mental health strategies in the post-pandemic recovery phase.

## 2 Methodology

The current study is a secondary analysis of a cross-sectional observational study carried out at Sher-i-Kashmir Institute of Medical Sciences (SKIMS) from January 2022 to December 2022. A consecutive sampling method was employed. All individuals meeting the inclusion criteria (PCR-confirmed COVID-19, ≥4 weeks post-clinical recovery, age ≥18 years) who attended the post-COVID outpatient follow-up clinic or were identified through community outreach during the study period were approached sequentially and invited to participate. No randomization or purposive selection based on symptom severity or demographic characteristics was applied. The primary methods and tools used in the study have been thoroughly detailed by our team previously (Sharief Banday et al., 2023). While the primary study comprehensively described prevalence estimates and sociodemographic correlates, the current study explores a novel research question which is associations between COVID-19 symptom features and psychological distress as measured by DASS-21, that was not examined in the primary analysis. We have minimized duplication of previously reported results by condensing sample description and prevalence data to a single summary sentence with a citation to the primary paper, allowing the present manuscript to focus on novel findings.

The methodology of the study was conducted in accordance with the principles outlined in the Declaration of Helsinki. In summary, 250 participants who met the inclusion criteria (the primary criterion being a PCR confirmed COVID-19 test result), including healthcare professionals and the general public, were recruited. Healthcare professionals (HCPs) were defined as individuals employed in clinical or paramedical roles at SKIMS (including doctors, nurses, technicians, and support staff) who had direct or indirect patient contact during the pandemic. The community group comprised non-healthcare individuals from the general population of the Kashmir Valley, recruited via community outreach. The data collection approach and the questionnaire have been outlined in our initial study. Briefly, all eligible participants were approached personally and invited to participate in the study. Participants were identified through outpatient follow-up clinics and community outreach; eligibility required PCR-confirmed COVID-19 and ≥4 weeks of clinical recovery. Written informed consent was obtained from all participants prior to enrollment.They were given a validated, self-designed structured questionnaire divided into three parts: socio-demographic details, COVID-19-related clinical parameters, and the DASS-21 scale. The selection of specific COVID-19 symptoms (fever, breathlessness, decreased level of consciousness, and duration of symptoms) was guided by prior literature identifying these as common manifestations of acute COVID-19 infection with potential prognostic significance.

The Depression Anxiety Stress Scale (DASS-21), developed by Lovibond and Lovibond, is a validated 21-item instrument that measures the core dimensions of depression, anxiety, and stress. Although it is not a diagnostic tool, it effectively assesses the severity of these emotional states. The DASS-21 has been extensively used and validated across various populations, with substantial research supporting its reliability and construct validity in both clinical and general populations (Beaufort et al., 2017). As per standard DASS-21 scoring guidelines, cutoff scores (multiplied by 2 to correspond to full DASS-42) were: Depression_normal (0–9), mild (10–13), moderate (14–20), severe (21–27), extremely severe (≥28); Anxiety_normal (0–7), mild (8–9), moderate (10–14), severe (15–19), extremely severe (≥20); Stress_normal (0–14), mild (15–18), moderate (19–25), severe (26–33), extremely severe (≥34).

Statistical analyses were performed using the Statistical Package for the Social Sciences (SPSS) software, version 20.0 (IBM Corp., Chicago, IL, USA). For the analysis of objectives of this very study we ran various statistical tests to address the specific aims of this study. Independent Samples *t*-test was used to determine if there was a statistically significant difference in the mean scores of depression, anxiety, and stress between participants who presented with a specific COVID-19 symptom (e.g., fever, breathlessness) and those who did not. It allowed us to identify which individual symptoms were associated with higher levels of psychological distress. For univariate analyses (Tables 2a-c and 3), we treated DASS-21 domain scores as continuous variables and used Independent Samples *t*-tests to compare mean scores between groups (e.g., fever present vs. absent). This approach preserves the full range of severity. We also compared the mean DASS-21 scores between two independent groups: participants with a longer duration of COVID-19 symptoms (≥15 days) and those with a shorter duration (<15 days), as well as those who were hospitalized and those who were not. The purpose was to evaluate whether prolonged illness or the need for inpatient care was linked to significantly higher levels of depression, anxiety, and stress.

To identify the most robust predictors of psychological distress while controlling for potential confounding effects among all variables, we performed a multivariable logistic regression. The dependent (outcome) variable was distress status, derived by dichotomizing DASS-21 scores according to established DASS-21 scores: participants with mild, moderate, severe, or extremely severe scores on any domain (depression, anxiety, or stress) were classified as distressed, while those with normal scores on all domains were classified as non-distressed. All variables listed in Tables 1, 2, and 3 (including age, gender, occupational status, monthly income, all 20 individual COVID-19 symptoms, symptom duration ≥15 days, and hospitalization status) were entered simultaneously into the model using the 'Enter' method. This approach was chosen a priori to adjust for all measured potential confounders, rather than using a stepwise selection procedure. This analysis provided Adjusted Odds Ratios (OR), which quantify the strength of association between each independent variable (e.g., a specific symptom) and the likelihood of experiencing significant psychological distress, after adjusting for all other variables in the model (e.g., the presence of other symptoms, duration of symptoms, hospital stay). A p-value <0.05 was considered statistically significant for all analyses.

**Table 1:** Sociodemographic profile of participants.

Variables		N = 250	Percent (%)
Gender	Male	101	40.4%
	Female	149	59.6%
Age (years)	18–25	77	30.8%
	26–35	76	30.4%
	36–45	52	20.8%
	>45	45	18.0%
Residence	Rural	116	46.4%
	Urban	134	53.6%
Marital Status	Married	138	55.2%
	Un-married	112	44.8%
Smoking Status	Non-Smoker	217	86.8%
	Ex-Smoker	10	4.0%
	Smoker	23	9.2%
Occupational Status HCP 161 (64.4%)	Doctor	37	14.8%
	Researcher	16	6.4%
	Nurse	25	10.0%
	Pharmacist	12	4.8%
	Allied Health Care Worker	24	9.6%
	Administrative Employee	19	7.6%
	Student	28	11.2%
Occupational Status Community 89 (35.6%)	Professional	24	9.6%
	Non-professional	11	4.4%
	Unemployed	54	21.6%
Monthly Income	< 50K	57	22.8%
	≥ 50K	120	48.0%
	No Income	73	29.2%

The Institutional Ethical Committee (IEC) of Sher-i-Kashmir Institute of Medical Sciences (Deemed Medical University) granted the approval to conduct the study (#RP 102/2022). All the participants’ details acquired in this study were kept confidential and no personal information of the study participants was disclosed in any form whatsoever.

## 3 Results

As previously reported for this cohort (Sharief Banday et al., 2023), the study sample consisted of 250 participants, predominantly female (59.6%), with a mean age of 34.28 years. The cohort was categorized into healthcare professionals (HCPs), comprising 161 individuals (64.4%), and community members, comprising 89 individuals (35.6%). Table 1 describes the overall study sample.

More than half of the HCPs experienced depression (54.8%), over two-thirds reported anxiety (68.0%), and slightly over a third had stress (34.4%). In the community cohort, 68.5% participants exhibited depressive symptoms, 74.1% anxious symptoms, and 38.2% stress symptoms. The DASS-21 questionnaire demonstrated high reliability (Overall α = 0.915; HCPs α = 0.923; Community α = 0.898), and significant intercorrelations were observed among depression, anxiety, and stress scores (p < 0.001). Furthermore, analysis of socio-demographic variables revealed that anxiety levels were significantly associated with specific occupational roles among healthcare professionals (p = 0.013), particularly affecting nurses, students, and doctors, while no other demographic factors showed a significant association with depression, anxiety, or stress scores.

Association of COVID-19 symptoms with depression, anxiety, and stress scores was performed using univariate analysis (Table 2a,b,c). Fever (80.8%), sore throat (68.8%), cough (66%), fatigue (65.6%), headache (64%), and myalgia (45.2%) were the most common symptoms reported by the majority of study subjects. In the univariate analysis model, symptoms such as a decreased level of consciousness were significantly associated with depression (p < 0.0248), anxiety (p < 0.0232), and stress (p < 0.0170) scores; however, breathlessness among all symptoms was found to show a very high significant association with depression (p < 0.0021), anxiety (p < 0.001), and stress (p < 0.0170) scores. Arthralgia was found to have a highly significant association (p < 0.0008) with stress scores. The remaining symptoms showed no significant correlation.

**Table 2a:** Association of Depression scores with COVID-19 symptoms using an Independent sample *t*-test.

**Symptoms**	Yes		No		**p-value**
	**N**	Mean±SD	**N**	Mean±SD	
Fever	202	12.03 ± 9.13	48	10.38 ± 11.27	0.2826
Breathlessness	98	14.02 ± 10.09	152	10.22 ± 8.94	0.0021⁎⁎
Cough	165	11.24 ± 9.04	85	12.64 ± 10.53	0.2746
Sore throat	172	11.62 ± 10.18	78	11.92 ± 8.14	0.8149
Fatigue	164	11.27 ± 10.19	86	12.56 ± 8.26	0.3125
Myalgia	113	11.66 ± 10.61	137	11.75 ± 8.67	0.9424
Arthralgia	33	14.12 ± 11.85	217	11.35 ± 9.15	0.1209
Rhinitis	58	12.52 ± 10.2	192	11.47 ± 9.39	0.4659
Headache	160	11.28 ± 9.1	90	12.49 ± 10.37	0.3369
Pain abdomen	23	12.43 ± 8.68	227	11.64 ± 9.67	0.7048
GERD	21	9.43 ± 7.62	229	11.92 ± 9.72	0.2542
Decreased level of Consciousness	21	16.19 ± 10.1	229	11.3 ± 9.44	0.0248*
Anosmia (Loss of Smell)	91	11.74 ± 9.51	159	11.7 ± 9.64	0.9759
Dysgusia (Altered Taste)	78	12.15 ± 8.88	172	11.51 ± 9.89	0.6241
Presyncope	13	14.92 ± 9.82	237	11.54 ± 9.55	0.2149
Nausea	42	10.71 ± 9.18	208	11.91 ± 9.66	0.4601
Vomiting	14	11.57 ± 4.71	236	11.72 ± 9.79	0.9550
Diarrhoea	29	12.97 ± 10.04	221	11.55 ± 9.52	0.4544
Skin Rash	12	13.67 ± 11.28	238	11.61 ± 9.5	0.4696
Haemoptysis	3	12.67 ± 1.15	247	11.7 ± 9.63	0.8625

SD, standard deviation.

⁎⁎ highly significant difference at *p* < 0.01;.

⁎ significant difference at *p* < 0.05.

**Table 2b:** Association of Anxiety scores with COVID-19 symptoms using an Independent sample *t*-test.

**Symptoms**	Yes		No		**p-value**
	**N**	Mean±SD	**N**	Mean±SD	
Fever	202	12.88 ± 8.93	48	10.38 ± 9.22	0.0836
Breathlessness	98	15.67 ± 9.75	152	10.29 ± 7.85	<0.001⁎⁎
Cough	165	12.32 ± 8.77	85	12.56 ± 9.53	0.8363
Sore throat	172	12.5 ± 9.32	78	12.18 ± 8.38	0.7952
Fatigue	164	12.54 ± 9.69	86	12.14 ± 7.62	0.7417
Myalgia	113	11.93 ± 9.07	137	12.79 ± 8.99	0.4547
Arthralgia	33	14.3 ± 9.67	217	12.11 ± 8.91	0.1939
Rhinitis	58	13.21 ± 9.41	192	12.16 ± 8.91	0.4381
Headache	160	12.11 ± 8.73	90	12.91 ± 9.54	0.5028
Pain abdomen	23	14.43 ± 10.13	227	12.19 ± 8.9	0.2571
GERD	21	10.38 ± 7.63	229	12.59 ± 9.13	0.2848
Decreased level of Consciousness	21	16.67 ± 8.28	229	12.01 ± 9	0.0232*
Anosmia (Loss of Smell)	91	12.77 ± 8.83	159	12.19 ± 9.15	0.6254
Dysgusia (Altered Taste)	78	12.72 ± 8.74	172	12.26 ± 9.17	0.7083
Presyncope	13	16.46 ± 10.46	237	12.18 ± 8.91	0.0955
Nausea	42	13.33 ± 9.41	208	12.21 ± 8.95	0.4634
Vomiting	14	10.85 ± 5.80	236	12.49 ± 9.17	0.5112
Diarrhoea	29	12.34 ± 9.26	221	12.41 ± 9.01	0.9721
Skin Rash	12	15.17 ± 11.65	238	12.26 ± 8.88	0.2771
Haemoptysis	3	12.00 ± 4.00	247	12.4 ± 9.07	0.9386

SD: standard deviation.

⁎⁎ highly significant difference at *p* < 0.01;.

⁎ significant difference at *p* < 0.05.

**Table 2c:** Association of Stress scores with COVID-19 symptoms using an Independent sample *t*-test.

**Symptoms**	Yes		No		**p-value**
	**N**	Mean±SD	**N**	Mean±SD	
Fever	202	12.37 ± 8.56	48	11.25 ± 11.08	0.4451
Breathlessness	98	14.31 ± 9.7	152	10.76 ± 8.4	0.0024⁎⁎
Cough	165	11.75 ± 8.67	85	12.94 ± 9.83	0.3251
Sore throat	172	12.64 ± 9.6	78	11.08 ± 7.77	0.2082
Fatigue	164	12.56 ± 9.81	86	11.37 ± 7.5	0.3265
Myalgia	113	12.99 ± 10	137	11.46 ± 8.23	0.1852
Arthralgia	33	17.03 ± 11.64	217	11.41 ± 8.42	0.0008⁎⁎
Rhinitis	58	13.83 ± 9.38	192	11.65 ± 8.95	0.1091
Headache	160	12.19 ± 9.17	90	12.09 ± 8.97	0.9345
Pain abdomen	23	13.39 ± 9.33	227	12.03 ± 9.07	0.4934
GERD	21	11.9 ± 8.04	229	12.17 ± 9.19	0.8966
Decreased level of Consciousness	21	16.67 ± 9.6	229	11.74 ± 8.94	0.0170*
Anosmia (Loss of Smell)	91	11.96 ± 9.43	159	12.26 ± 8.9	0.7969
Dysgusia (Altered Taste)	78	13 ± 9.48	172	11.77 ± 8.9	0.3212
Presyncope	13	15.23 ± 10.6	237	11.98 ± 8.99	0.2101
Nausea	42	12.67 ± 9.39	208	12.05 ± 9.04	0.6881
Vomiting	14	10.43 ± 6.47	236	12.25 ± 9.21	0.4661
Diarrhoea	29	13.10 ± 9.50	221	12.03 ± 9.04	0.5496
Skin Rash	12	13.33 ± 10.39	238	12.09 ± 9.03	0.6452
Haemoptysis	3	14.00 ± 4.00	247	12.13 ± 9.13	0.7237

SD: standard deviation.

⁎⁎ highly significant difference at p < 0.01;.

⁎ significant difference at p < 0.05.

Association of duration of COVID-19 symptoms duration and hospital stay with depression, anxiety, and stress scores using univariate analysis is shown in Table 3. Duration of COVID-19 symptoms showed a highly significant association with depression (p < 0.007), stress (p < 0.006), and a significant association with anxiety (p < 0.010) scores. However, no significant association was found with hospital stay.

**Table 3:** Association of Depression, Anxiety, and Stress scores with duration of COVID-19 symptoms and hospital stay using an Independent sample *t*-test.

**Parameter**	**DASS score**	**Category**	**N**	**Mean**	**Std. Deviation**	**p-value**
Symptom days	Depression score	<15 days	213	11.0329	9.11270	0.007⁎⁎
		≥ 15 days	37	15.6216	11.24315	
	Anxiety score	<15 days	213	11.7934	8.89109	0.010*
		≥15 days	37	15.8919	9.09146	
	Stress score	<15 days	213	11.5023	8.72841	0.006⁎⁎
		≥15 days	37	15.8919	10.25179	
Hospital stay	Depression score	Not Hospitalized	234	11.4615	9.37904	0.114
		Hospitalized	16	15.3750	11.81454	
	Anxiety score	Not Hospitalized	234	12.2393	8.96407	0.282
		Hospitalized	16	14.7500	9.82174	
	Stress score	Not Hospitalized	234	12.0342	9.02636	0.434
		Hospitalized	16	13.8750	10.02580	

⁎⁎ highly significant difference at p < 0.01.

⁎ significant difference at p < 0.05.

For multivariable analysis (Table 4), DASS-21 scores were dichotomized to create a binary distress outcome (distressed vs. non-distressed) based on established standard DASS-21 scores (mild to extremely severe in any domain = distressed). Each clinical variable (symptom, prolonged duration, hospitalization) was entered as a binary predictor (present/absent or yes/no). Fever (OR=4.284, p = 0.001, 95% CI=1.812–10.127) and breathlessness (OR=3.223, p = 0.003, 95% CI=1.505–6.905) showed a highly significant association with DASS-21 scores. In addition, decreased level of consciousness (OR=11.443, p = 0.034, 95% CI=1.198–109.29) and dysgusia/altered taste (OR=2.777, p = 0.54, 95% CI=0.983–7.846) were significantly associated with DASS-21 scores. No significant correlation was found with the rest of the symptoms, duration of COVID-19 symptoms, or hospital stay.

**Table 4:** Multivariable Logistic Regression Analysis of Depression, Anxiety, and Stress (DASS-21) scores with respect to various variables.

**Factor**	**Standard Error**	**p-value**	**Adjusted Odds Ratio**	**95% C.I. for Odds Ratios**	
				**Lower**	**Upper**
Fever(1)	.439	.001⁎⁎	4.284	1.812	10.127
Breathlessness(1)	.389	.003⁎⁎	3.223	1.505	6.905
Cough(1)	.382	.346	.697	.330	1.476
Sore throat(1)	.406	.618	.817	.369	1.808
Fatigue(1)	.434	.149	.534	.228	1.251
Myalgia(1)	.393	.086*	.509	.236	1.100
Arthralgia(1)	.550	.339	1.693	.576	4.977
Rhinitis(1)	.391	.386	.712	.331	1.534
Headache(1)	.365	.677	1.164	.569	2.382
Pain abdomen(1)	.740	.578	1.510	.354	6.432
GERD(1)	.736	.436	.564	.133	2.387
Decreased level of consciousness(1)	1.151	.034*	11.443	1.198	109.29
Anosmia/loss of smell(1)	.490	.181	.519	.199	1.357
Dysgusia/altered taste(1)	.530	.054*	2.777	.983	7.846
Presyncope(1)	.840	.600	1.553	.299	8.064
Nausea(1)	.482	.694	.828	.322	2.128
Vomiting(1)	1.141	.159	4.985	.532	46.69
Diarrhoea(1)	.516	.478	.693	.252	1.905
Skin rash(1)	.861	.855	.854	.158	4.617
Haemoptysis(1)	1.498	.661	.518	.027	9.758
Duration of symptoms(1*)	.521	.562	1.352	.487	3.752
Hospital stay(1)	.711	.653	.726	.180	2.928

(1)=Yes/No; (1*)= ≥15 days/<15 days.

⁎⁎ highly significant difference at p < 0.01.

⁎ significant difference at p < 0.05.

## 4 Discussion

Our research group in the primary study measured the psychological distress caused among the individuals who had tested positive for COVID-19 using the DASS-21. In this secondary analysis, we assessed the association of various variables, such as COVID-19 symptoms, their duration, and hospital stay, with depression, anxiety, and stress scores. Excluding the normal study subjects, the results in the primary study show that 54.8%, 68%, and 34.4% participants experienced depression, anxiety, and stress, respectively. Among the HCPs, the maximum number of participants reported extremely severe anxiety (21.1%), moderate depression (15.5%), and mild and moderate stress each (11.8%). Contrarily, the majority of the study subjects in the community had moderate depression (33.7%), moderate anxiety (23.6%), and moderate stress (16.9%) (Sharief Banday et al., 2023). These findings corroborate similar studies conducted in India (Ray, 2022; Verma and Mishra, 2020; Sharma et al., 2022; Abdulla et al., 2021; Kumar and Kumar 2020). Our study cohort consisted exclusively of Kashmiris, Kashmir being a conflict-torn region for decades, has faced constant challenges, including an identity crisis, socio-economic hurdles, thus the population here has long been exposed to psychological stressors (Connah, 2021; Amin and Khan, 2009). In such a context, COVID-19 was expected to worsen the fragile psychological framework among an already vulnerable population. Previous studies support this notion, affirming that individuals with frail psychological resilience are at a greater risk for adverse COVID-19-related mental health outcomes (Malik et al., 2022).

The elevated prevalence of psychological morbidity observed in this study are a consequence of multiple interrelated factors. The widespread economic instability, unemployment, and social restrictions that accompanied lockdowns profoundly disrupted daily routines, education, and occupational security. These changes, compounded by social isolation, loneliness, and the pervasive fear of infection, likely are associated with heightened emotional distress. Moreover, the social stigma associated with confirmed COVID-19 infection and quarantine, particularly during the early stages of the pandemic, may have intensified psychological strain and feelings of alienation (Wang et al., 2020). Such multifaceted stressors collectively underscore the broad psychosocial toll of the pandemic across both professional and community populations.

Using univariate and multivariable regression analysis, in this study, we identified a few symptoms presented by participants after they tested COVID-positive, which were associated with depression, anxiety, and stress prevalence measured by DASS-21. Breathlessness and a decreased level of consciousness showed a statistically highly significant association with depression, anxiety, and stress. Arthralgia showed a significant association with stress only. Multivariable regression analysis showed fever (OR=4.2, 95% CI: 1.8–10.1), myalgia (OR=0.8, 95% CI: 0.2–1.1), dysgusia/altered taste (OR=0.5, 95% CI: 0.9–7.8), breathlessness (OR=3.2, 95% CI: 1.5–6.9), decreased level of consciousness (OR=11.4, 95% CI: 1.1–109.2), statistically significant symptoms associated with prevalence of depression, anxiety and stress. The findings of other studies also revealed that physical symptoms are associated with psychological distress (Varshney et al., 2020; He et al., 2021). The plausible explanation for these associations lies in the perceived threat linked to specific symptoms. Fever, myalgia, and breathlessness, being hallmark features of severe COVID-19 pneumonia (Ng et al., 2021), may have evoked fear of clinical deterioration, hospitalization, and death. The association between fever and psychological distress may be explained by both biological and psychosocial mechanisms. Fever is a prominent, easily recognized symptom often linked in public perception to severe infection and risk of deterioration. Culturally, in the Kashmir region, fever is frequently associated with serious illness, which may amplify anxiety. Biologically, fever-induced systemic inflammation can affect the central nervous system, potentially contributing to mood disturbances. However, our data do not allow us to disentangle these mechanisms, and this remains an important area for future research. Similarly, uncommon symptoms such as decreased level of consciousness and arthralgia might have amplified uncertainty about the disease severity and prognosis, thereby augmenting the psychological distress (Wang et al., 2020). Studies have highlighted how pre-existing co-morbid conditions like diabetes, hypertension, and respiratory disorders can aggravate COVID-19-related symptoms, including breathlessness, chills, fever, body pains, and many others, potentially progressing the disease to spiral out of treatment protocol and, in extreme scenarios, culminating in death (Ahmad Malik et al., 2022; Malik et al., 2022). This interplay between somatic manifestations and psychological reactions thus appears to be a critical pathway through which COVID-19 affected mental health.

In addition, the duration of symptoms demonstrated a significant association with depression, anxiety, and stress, with higher scores observed among participants who experienced symptoms for ≥15 days. Prolonged illness likely extended the period of isolation, uncertainty, and disruption to daily functioning, thereby exacerbating psychological burden. Previous research supports this finding, suggesting that quarantine-related factors such as fear, loneliness, and boredom significantly influence mental health outcomes (Brooks et al., 2020).

Conversely, hospital stay did not exhibit a statistically significant relationship with DASS-21 scores in the present study. This contrasts with reports from similar investigations where hospitalization was associated with greater psychological distress (Ahmed et al., 2022). One possible explanation for this discrepancy is the relatively small proportion of hospitalized participants (6.4%) in our cohort, which may have limited statistical power. Additionally, improvements in hospital care, patient communication, and virtual social connectivity during later pandemic waves might have mitigated the psychological burden of hospitalization.

Overall, the findings from this study highlight the intricate interrelationship between physical symptoms and psychological responses associated with COVID-19 infection. The observed associations underscore the need for an integrated approach to patient care that encompasses both physical and mental health dimensions. Routine mental health screening using standardized tools such as DASS-21 should be incorporated into COVID-19 management protocols, particularly for individuals exhibiting severe or prolonged symptoms. Moreover, early psychosocial interventions, including counseling, peer support, and neuropsychiatry services, should be prioritized to prevent long-term mental health sequelae.

Moreover, our findings have direct implications for healthcare system planning in Kashmir and similar low-resource settings. First, the high prevalence of distress among HCPs (54.8% depression, 68% anxiety) threatens workforce retention and patient safety. Integration of routine mental health screening into occupational health programs is urgently needed. Second, the association of specific symptoms (fever, breathlessness, prolonged illness) with distress suggests that tele-psychiatric follow-up for high-risk COVID-19 survivors could be cost-effective. Third, training primary care providers to recognize and address psychological distress in post-COVID patients should be incorporated into continuing medical education. Without such measures, the region faces a dual burden: untreated mental illness and HCPs’ burnout.

## 5 Conclusion

Our study not only revealed the substantial prevalence of depression, anxiety, and stress in Kashmiri population due to COVID-19, but we also identified several risk factors associated with depression, anxiety, and stress prevalence such as symptoms of fever, breathlessness, arthralgia, myalgia, dysgusia, and decreased level of consciousness, and duration of symptoms (≥15 days or < 15 days). These risk factors should be considered potential indicators for targeted mental health screening for COVID-19 patients. Identifying individuals with these risk profiles can facilitate early intervention to alleviate distress. These findings underscore the urgent need to integrate mental health support into pandemic response and recovery plans, transforming the "silent pandemic" of psychological distress into an addressed priority.

## 6 Limitations

Several limitations warrant consideration. The cross-sectional design precludes causal inference and allows for reverse causation (distress influencing symptom recall). Generalizability is limited by the over-representation of healthcare workers (64.4%) and a single-region sample (Kashmir). Key confounders not measured in the primary dataset include prior mental health history, domestic violence, substance misuse (beyond smoking), and oxygen requirements/ICU admission. Dichotomization of DASS-21 scores into a binary distress outcome for multivariable analysis, while clinically meaningful for generating interpretable odds ratios, reduces statistical power compared to continuous analysis. Therefore, we have presented both continuous univariate analyses (Tables 2a-c, 3) and dichotomized multivariable findings (Table 4) to provide a comprehensive assessment. Finally, recall bias may affect symptom reporting as surveys were completed post-recovery, and the small number of hospitalized participants (n = 16) limited power to detect hospitalization effects.

## CRediT authorship contribution statement

**Mutha Manzoor:** Writing – review & editing, Writing – original draft, Data curation, Conceptualization. **Muddasir Sharief Banday:** Writing – review & editing, Supervision, Project administration, Methodology. **Bilal Ahmad Para:** Writing – review & editing, Validation, Software, Formal analysis. **Vanitha Innocent Rani:** Writing – review & editing, Project administration, Investigation, Funding acquisition. **Umme Hani:** Writing – review & editing, Project administration, Methodology, Funding acquisition. **Subi Mol Raja:** Writing – review & editing, Investigation, Formal analysis, Data curation. **Sumaila Parveen:** Writing – review & editing, Validation, Supervision, Resources. **S. Ilambharathi:** Writing – review & editing, Software, Methodology, Investigation. **M Kiruthika:** Writing – review & editing, Software, Methodology, Formal analysis. **Jonaid Ahmad Malik:** Writing – review & editing, Resources, Investigation, Formal analysis.

## Declaration of competing interest

None to declare.

## Data Availability

Data will be made available on request.
